# Functional analysis of lipid metabolism genes in wine yeasts during alcoholic fermentation at low temperature

**DOI:** 10.15698/mic2014.11.174

**Published:** 2014-10-29

**Authors:** María López-Malo, Estéfani García-Ríos, Rosana Chiva, José M. Guillamon

**Affiliations:** 1 Departamento de Biotecnología de los alimentos, Instituto de Agroquímica y Tecnología de los Alimentos (CSIC), Avda, Agustín Escardino, 7, E-46980-Paterna, Valencia, Spain.; 2 Biotecnologia Enològica. Departament de Bioquímica i Biotecnologia, Facultat d’Enologia, Universitat Rovira i Virgili, Marcel•li Domingo s/n, 43007, Tarragona, Spain.

**Keywords:** wine, industrial yeast, cold adaptation, lipids, mutant, stable overexpression, fermentation

## Abstract

Wine produced by low-temperature fermentation is mostly considered to have improved sensory qualities. However few commercial wine strains available on the market are well-adapted to ferment at low temperature (10 - 15°C). The lipid metabolism of *Saccharomyces cerevisiae* plays a central role in low temperature adaptation. One strategy to modify lipid composition is to alter transcriptional activity by deleting or overexpressing the key genes of lipid metabolism. In a previous study, we identified the genes of the phospholipid, sterol and sphingolipid pathways, which impacted on growth capacity at low temperature. In the present study, we aimed to determine the influence of these genes on fermentation performance and growth during low-temperature wine fermentations. We analyzed the phenotype during fermentation at the low and optimal temperature of the lipid mutant and overexpressing strains in the background of a derivative commercial wine strain. The increase in the gene dosage of some of these lipid genes, e.g., *PSD1*, *LCB3, DPL1* and *OLE1,* improved fermentation activity during low-temperature fermentations, thus confirming their positive role during wine yeast adaptation to cold. Genes whose overexpression improved fermentation activity at 12°C were overexpressed by chromosomal integration into commercial wine yeast QA23. Fermentations in synthetic and natural grape must were carried out by this new set of overexpressing strains. The strains overexpressing *OLE1* and *DPL1* were able to finish fermentation before commercial wine yeast QA23. Only the *OLE1* gene overexpression produced a specific aroma profile in the wines produced with natural grape must.

## INTRODUCTION

Temperature is one of the most important parameters to affect the length and rate of alcoholic fermentation and final wine quality. Many winemakers prefer low-temperature fermentation (10 - 15°C) for the production of white and rosé wine because it improves taste and aroma characteristics. This improved quality can be attributed not only to the prevention of volatilization of primary aromas, but also to the increased synthesis of secondary aromas. Thus the final wine possesses greater terpenes retention, reduced higher alcohols and an increased proportion of ethyl and acetate esters in the total volatile compounds 1,2,3,4. Another positive aspect is that low temperatures reduce the growth of acetic and lactic bacteria, thus making it easier to control alcoholic fermentation.

Despite low-temperature fermentations offering interesting improvements, this practice also has its disadvantages. The optimal growth and fermentation temperature for *Saccharomyces cerevisiae* is 25 - 28°C. Restrictive low temperature increases the lag phase and lowers the growth rate, leading to sluggish and stuck fermentations 5. Therefore, the quality of those wines produced at low temperature depends on the yeast’s ability to adapt to cold.

The importance of lipid composition in the yeast adaptive response at low temperature is well-known 1,4,6,7. A drop in temperature leads to diminished membrane fluidity 8. To counteract this membrane rigidity, yeasts were able to develop several mechanisms to maintain appropriate fluidity. The most commonly studied involves increased unsaturation and reduced average chain length of fatty acids (FA) 1,4. Recently, 7 also reported new common changes in the lipid composition of different industrial species and strains of *Saccharomyces* after growth at low temperature. Despite specific strain-/species-dependent responses, the results showed that the medium chain FA and triacylglyceride content increased at low temperatures, whereas phosphatidic acid content and the phosphatidylcholine/phosphatidylethanolamine (PC/PE) ratio decreased. In this way, cells can also be influenced by the environment during wine fermentation because yeast can incorporate fatty acids from the medium into its own phospholipids 1,9. In grapes, unsaturated fatty acids represent the major component of total lipids. The most abundant is linoleic acid (C18:2), followed by oleic (C18:1), linolenic (C18:3) and palmitoleic acid (C16:1) 10.

In *S. cerevisiae*, these metabolic changes are primarily governed by the regulation of the transcriptional activity of those genes involved in the lipid biosynthesis pathway. Tai *et al*. 11 compared different genome-wide transcriptional studies of *S. cerevisiae* grown at low temperature. They concluded that the lipid metabolism genes were the only ones whose activity was clearly regulated by low temperature. In a recent work, we also demonstrated that the main differences between the metabolic profiling of *S. cerevisiae *growing at 12°C and 28°C were related to lipid metabolism 12. In another study by our group, we also screened the importance of most of the genes belonging to the phospholipid, sterol and sphingolipid pathways in adaptation to low temperature by analyzing the effect on growth in a laboratory and an industrial strain 13. From this previous study, the genes whose deletion and overexpression showed the greatest effect on growth were the following: *PSD1, CHO2 *and* OPI3, *of the phospholipid metabolism;* ERG3, ERG6 *and* IDI1, *of the ergosterol pathway; *LCB3, LCB4 *and* DPL1, *belonging to the sphingolipid pathway; and *OLE1*, the only desaturase of *S. cerevisiae*. The aim of the present study was to conduct an in-depth study of these selected genes in a context that mimicked wine fermentation conditions. Firstly, we analyzed the gene activity of the selected genes in several low-temperature fermentations of synthetic grape must in wild-type and overexpressing strains. We then characterized the effect of the mutations and overexpressions in a derivative wine strain on growth and fermentation activity in wine fermentations at low and optimum temperature. The increase in the gene dosage of some of these lipid genes improved both growth and fermentation activity in low-temperature fermentations, thus confirming their positive role during wine yeast adaptation at low temperature. Finally, the genes that showed an improved phenotype were overexpressed by integrating one or more copies in the delta regions of the genome of commercial wine yeast QA23 14. These stable overexpressing strains were retested in synthetic and natural grape must fermentations. The fermentative aroma compounds obtained in these wines were also analyzed.

## RESULTS

### Gene expression

#### Expression of the selected genes during fermentations at 12°C vs. 28°C

**Figure 1:**
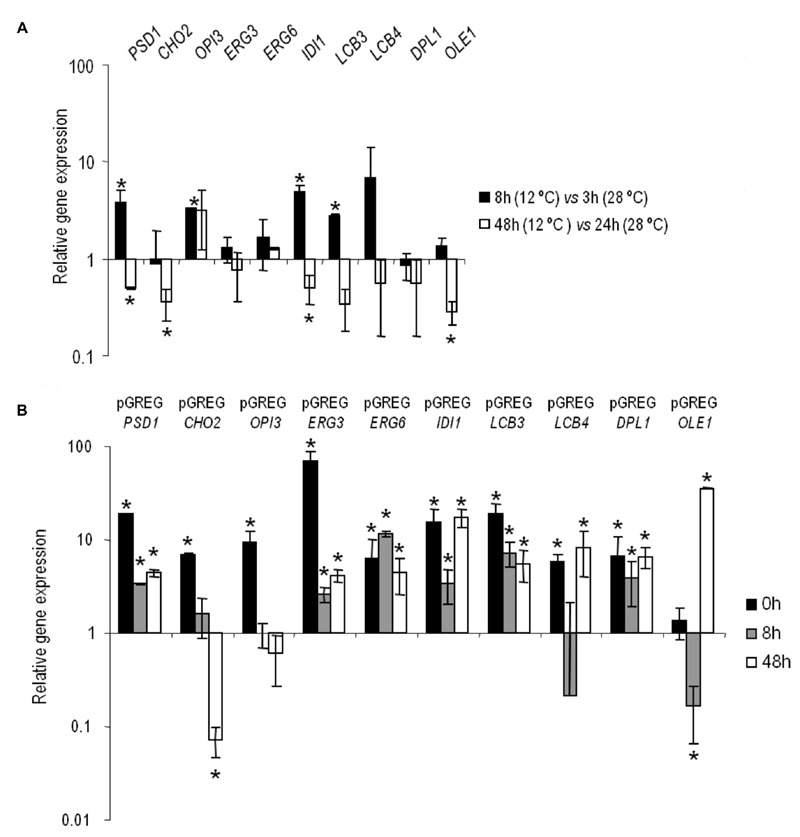
FIGURE 1: (A) Relative expression of the selected genes in the haploid strain *ho*QA23 at different stages of alcoholic fermentation. The gene expression in the lag and exponential phases of fermentation at 12°C are shown as a relative value in comparison to the control fermentation at 28°C. Values over 1 indicate higher gene expression at 12°C, whereas those under 1 indicate higher gene expression at 28°C. (B) Relative expression of the overexpressed genes. The differences in gene expression in the selected overexpressing strains are shown in relation to control *ho*QA23-pGREG (set as value 1). Values over 1 indicate higher gene expression than the control, whereas values under 1 indicate lower gene expression in comparison to the control. *The results with statistically significant differences (P-value ≤ 0.05).

The changes in the gene expressions at low temperature of *PSD1*, *CHO2*, *OPI3, ERG3*, *ERG6, IDI1*, *LCB3*, *LCB4*, *DPL1* and *OLE1* were analyzed in the control *ho*QA23 strain during the first fermentation stages at 12°C and 28°C. Prior to taking samples, growth curves were analyzed to select the hours corresponding to the lag and exponential phases at both temperatures (the same OD in both curves). Thus samples were taken in the lag (3 h at 28°C and 8 h at 12°C) and exponential phases (24 h at 28°C and 48 h at 12°C) during fermentation. The relative gene expression is shown in Figure 1A. The values higher and lower than 1 indicate a higher and lower gene expression at 12°C in comparison to 28°C. Save very few exceptions, these lipid genes showed higher activity during the lag or adaptation period at low temperature and, conversely, they were more active in the exponential phase at 28°C.

#### Verification of the overexpression in haploid wine strain hoQA23

Having determined gene activity at both temperatures in the key wine fermentation phases, we aimed to validate and quantify the overexpression of the constructed strains. Samples were taken before inoculation (time 0) and at the same time points (8 h and 48 h) at low temperature. The relative gene expression values of the overexpressing strains, normalized with the values of the control haploid strain (*ho*QA23-pGREG), are shown in Figure 1B. All the constructed strains presented increased overexpressed gene activity, which ranged from 3.5- to 68-fold more than the control strain at most of the time points analyzed. Thus we can verify that the constructed strains overexpressed the gene of interest.

### Phenotypic analysis of the mutant and overexpressing strains of *ho*QA23

#### Determination of generation time (GT) during growth in a synthetic grape must (SM)

**Figure 2:**
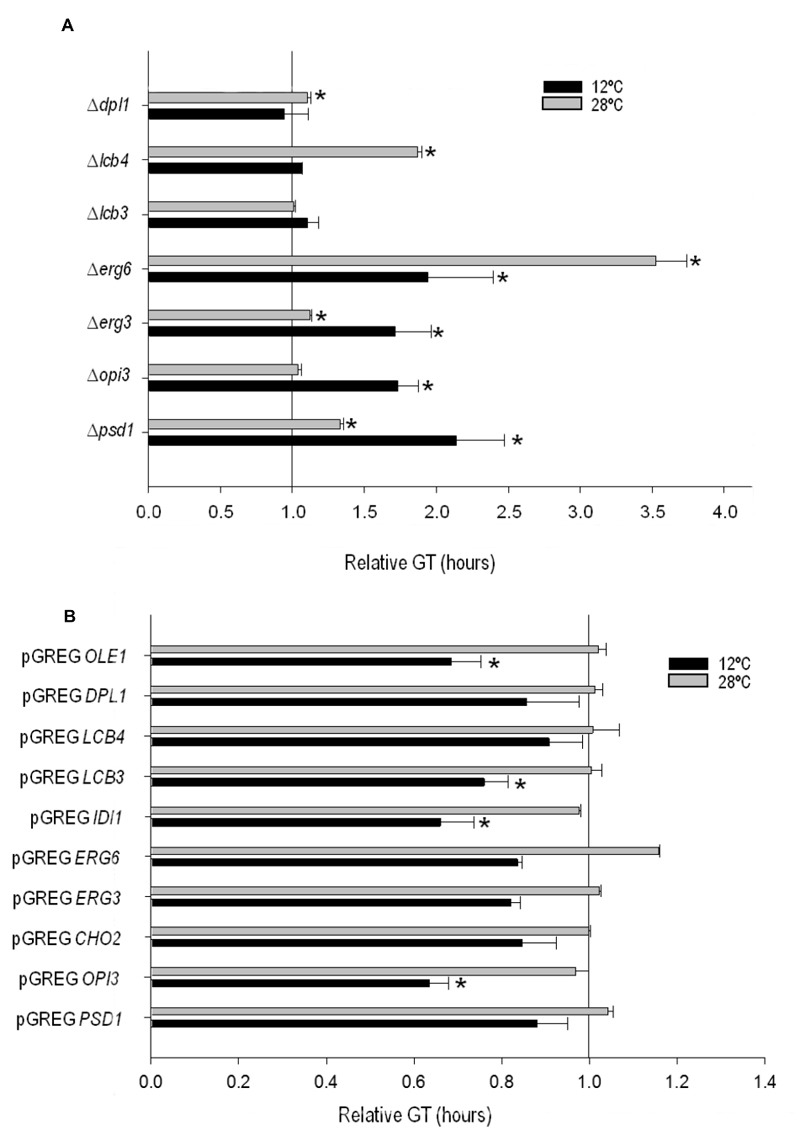
FIGURE 2: Generation time (GT) of (A) the mutant and (B) selected overexpressing strains grown at 12°C (black bars) and at 28°C (gray bars) normalized with the GT of their control strains *ho*QA23 and *ho*QA23-pGREG (normalized as value 1). The GT for the control strains was as follows: 11.59 h ± 3.12 h and 3.48 h ± 0.06 h for *ho*QA23 and 13.83 h ± 0.05 h and 3.63 h ± 0.05 h for *ho*QA23-pGREG at 12°C and 28°C, respectively. *Statistically significant differences (P-value ≤ 0.05) if compared with the control strain at the same temperature.

In order to determine the importance of the deletion or overexpression of the selected genes on growth at low temperature in wine fermentation, we calculated the GT of the mutant and overexpressing strains at 12°C and 28°C in SM (Fig. 2). All the phospholipid and sterol mutants showed worse growth than the control strain at 12°C, whereas no significant differences were observed for the sphingolipid mutants at this temperature. The GT of several mutants also increased at 28°C if compared with *ho*QA23. However, the differences were much larger at 12°C than at 28°C in ∆psd1 and ∆*erg3*.

Likewise, the GT of the overexpressing strains was also determined (Fig. 2). Most of the overexpressing strains showed a substantially shorter GT at low temperature, although significant differences were noted only for pGREG *OPI3*, pGREG *IDI1*, pGREG *LCB3* and pGREG *OLE1*.

#### Fermentation activity of the mutant and overexpressing strains of hoQA23

**Figure 3:**
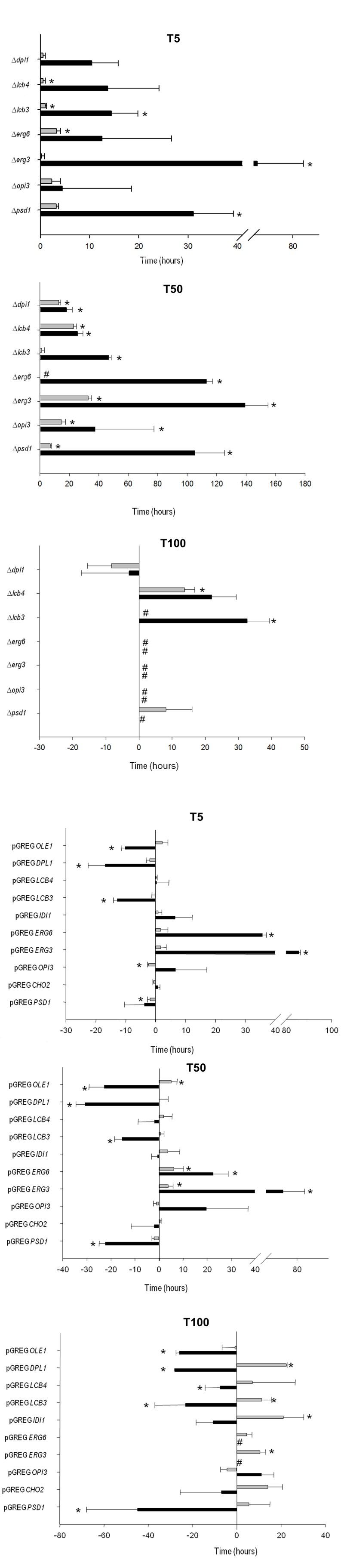
FIGURE 3: Determination of the time required by the mutant and the selected overexpressing strains to ferment 5% (T5), 50% (T50) and 100% (T100) of the initial sugar content in SM at 12°C (black bars) and 28°C (gray bars). Positive and negative values respectively represent the increases and decreases in time (hours) of the mutant and the overexpressing strains if compared to the control strains (normalized as value 0). The fermentation time of the control strains are: *ho*QA23 at 12°C T5 = 27 h ± 3.18 h, T50 = 96.19 h ± 3.97 h T100 = 251.44 h ± 10.34 h; at 28°C T5 = 6.23 h ± 0.93 h, T50 = 44.95 h ± 0.93 h, T100 = 131.14 h ± 2.32 h and *ho*QA23-pGREG at 12°C T5 = 41.63 h ± 7.16 h, T50 = 119.81 h ± 11.93 h T100 = 271.69 h ± 21.48 h; at 28°C T5 = 8.49 h ± 0.64 h, T50 = 38.40 h ± 1.56 h, T100 = 121.73 h ± 3.36 h. # Indicates stuck fermentation before T50 or T100. * Indicates statistically significant differences (P-value ≤ 0.05).

The fermentation kinetics of the mutant and overexpressing strains were estimated by calculating the time required to ferment 5% (T5), 50% (T50) and 100% (T100) of the sugars in the SM (Fig. 3). T5, T50 and T100 approximately match the beginning (lag phase), middle (end of the exponential phase) and end of fermentation, respectively. It should be highlighted that parental *ho*QA23 (control strain) and the same strain transformed with empty vector pGREG (the control strain of the overexpressing strains) showed differences in the T5, T50 and T100 (data provided in the figure legend). These differences may be explained by the presence of geneticin in the fermentations of the overexpressing strains and their resistance to this antibiotic encoded in the plasmid.

Deletion of some genes impaired the low-temperature fermentation performance of the wine strain. This was especially remarkable for ∆*psd1* and ∆*erg3,* which were significantly delayed at the beginning of the process (T5) (more than 30 h and 60 h, respectively). The ∆*psd1*, ∆*opi3*, ∆*erg3* and ∆*erg6 *mutant strains also needed more time to ferment 50% of the sugars (T50) and did not finish the fermentation process at low temperature. Although not as long, a similar delay in fermentation was also observed at 28°C for the ∆*opi3, *∆*erg3* and ∆*erg6* strains, but not for ∆*psd1*. This strain was considerably affected at low temperature, but was not affected at all at 28°C. Deletion of genes ∆*lcb4* and ∆*lcb3* affected the fermentation capacity at both low and optimum temperature*.* The latter gene deletion produced a stuck fermentation at 28°C.

Conversely, several overexpressing strains showed quicker fermentation activity at low temperatures. The overexpressions of *OLE1*, *DPL1* and* LCB3 *resulted in a shorter T5, T50 and T100. Despite pGREG *PSD1* did not start fermentation before the control, this strain displayed greater fermentation activity at T50 and finished almost 2 days before the fermentation if compared with the control *ho*QA23-pGREG strain. However, the overexpressions of *ERG3 *and* ERG6* resulted in a serious delay throughout the fermentation process at 12°C. pGREG *ERG3* and pGREG* ERG6* obtained longer T5 and T50, and were unable to finish fermentation. Interestingly, the overexpressions of *PSD1 *and *OLE1 *had no effect on fermentation length at 28°C.

### Stable overexpression of the selected genes in commercial wine yeast QA23

**Figure 4:**
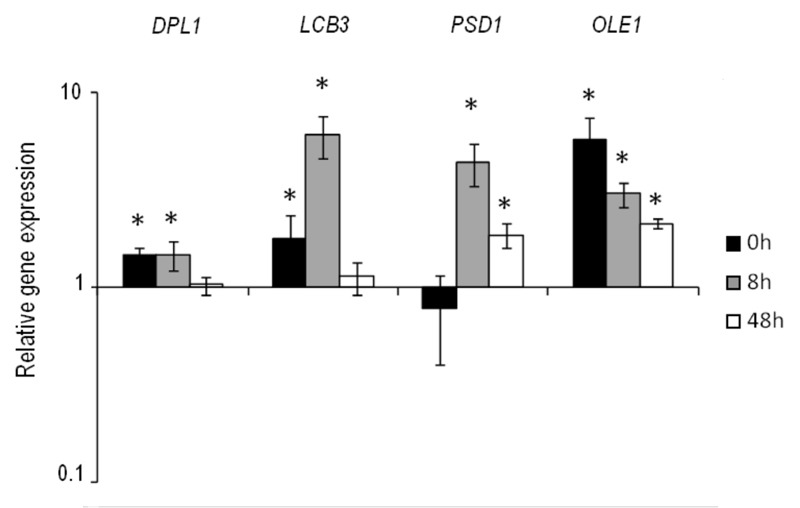
FIGURE 4: Relative expression of the overexpressed genes in the commercial wine yeast QA23. The differences in gene expression in the selected overexpressing strains are shown in relation to control QA23 (set as value 1). Values over 1 indicate higher gene expression than the control, whereas values under 1 indicate lower gene expression in comparison to the control. *The results with statistically significant differences (P-value ≤ 0.05).

Based on the previous results, we selected the four genes *DPL1*,* LCB3*, *OLE1 *and *PSD1* to construct stable overexpressing strains in the genetic background of the commercial wine yeast QA23. These copies were integrated by homologous recombination into the repetitive delta elements of Ty1 and Ty2. The correct integration of one or more copies was verified by PCR with primers homologous to the δ sequences. The overexpression of these strains was verified during wine fermentation in natural "Parellada" grape must at low temperature. The relative gene expression values were normalized with the commercial wine strain QA23 values (Fig. 4.). The four strains showed an overexpression of the target genes but, in all cases, the level of overexpression was lower than in the overexpressing strains of *ho*QA23, constructed by transformation with centromeric plasmids.

**Figure 5:**
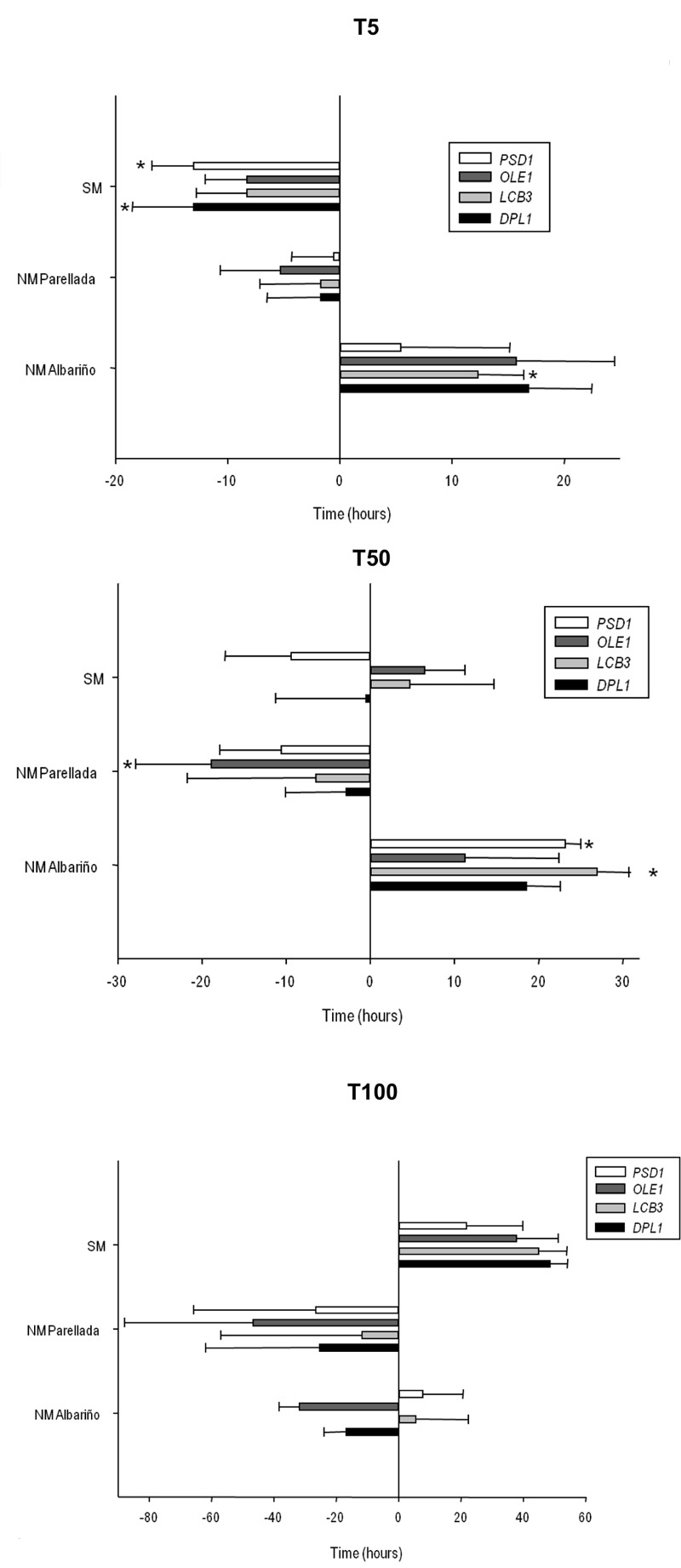
FIGURE 5: Determination of the time required by the stable overexpressing strains of commercial wine yeast QA23 to ferment 5% (T5), 50% (T50) and 100% (T100) of the initial sugar content in a SM, NM "Parellada" and NM "Albariño". Positive and negative values respectively represent the increases and decreases in time (hours) of the overexpressing strains if compared to the control strain (normalized as value 0). The fermentation time of the control strain, QA23, are in SM T5 = 70.36 h ± 5.34 h, T50 = 192.08 h ± 10.44 h, T100 = 383.86 ± 31.51 h; in NM "Parellada" T5 = 65.02 h ± 6.17 h, T50 = 190.89 h ± 1.02 h, T100 = 440.86 h ± 7.76 h and in NM "Albariño" T5 = 27 h ± 0.80 h, T50 = 104.06 h ± 3.18 h T100 = 266.63 h ± 26.25 h. * Indicates statistically significant differences (P-value ≤ 0.05).

These stable overexpressing strains were used to ferment both the SM and natural must (NM) of two different grape varieties (Albariño and Parellada). Yeast growth during fermentations was similar between the overexpressing strains and the commercial QA23 (data not shown). Minor differences were observed in the density reduction in the fermentations of both SM and NM carried out by the overexpressing strains as compared to that performed by commercial wine strain QA23 (Fig. 5). The overexpression of δ*DPL1*δ and δ*PSD1*δ resulted in a shorter T5 in the fermentations performed in SM, but no difference was found at the end of fermentation. Only δ*OLE1*δ was able to ferment 50% of sugars faster than the control in the "Parellada" grape fermentations. Moreover, δ*OLE1*δ and δ*DPL1*δ finished the fermentation process more quickly than QA23 in both "Parellada" and "Albariño" NM grape must fermentations.

**Figure 6:**
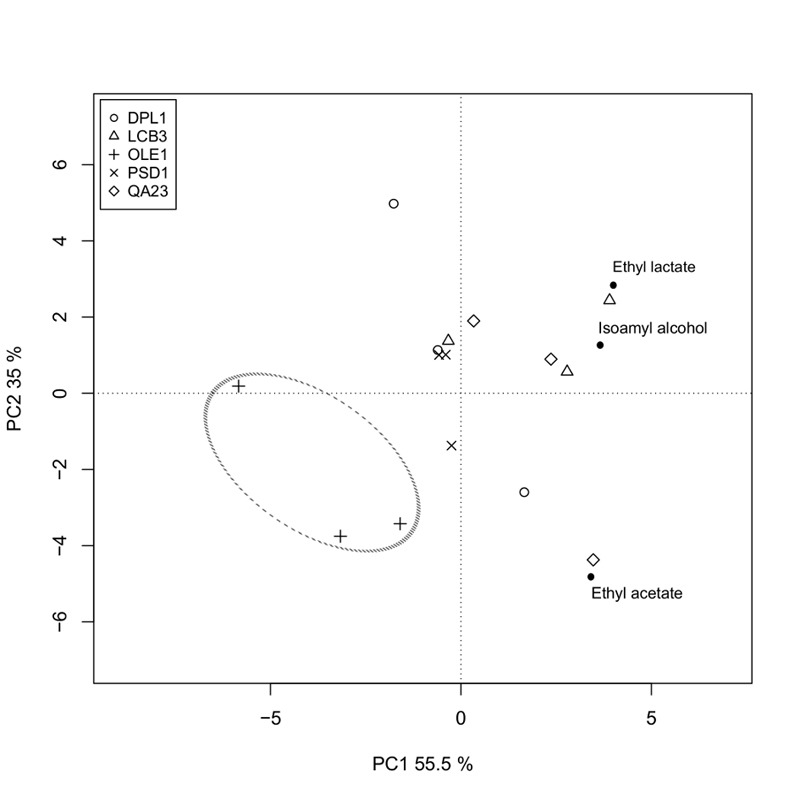
FIGURE 6: Biplot of the two first components of the principal component analysis according to the aroma composition of the selected overexpressing strains of QA23 in "Albariño" grape must at 12°C. Open symbols represent the samples and filled circles the aroma compounds with higher loadings.

We also analyzed the fermentative aroma compounds (higher alcohols, acetate esters and ethyl esters) in the wines obtained with both the overexpressing strains and the commercial wine yeast QA23 in "Albariño" grape must. A principal component analysis was performed to explore the effect of the overexpression of these genes on aroma composition (Fig. 6). The two first components were retained and explained 90.5% of total variance. The first principal component (PC1) accounted for 55.5% of total variance and was marked by high components loadings for ethyl lactate (+0.614) and isoamyl alcohol (+0.557). The second component loading explained 35% of the variation and was marked by high positive component loadings for ethyl lactate (+0.510) and isoamyl alcohol (+0.230), and by a high negative loading for ethyl acetate (-0.823).

The δ*OLE1*δ strain clearly separated from the other strains in the down-left quadrant, denoting the most specific aroma profile. The wine produced by this strain was the poorest in isoamyl alcohol, ethyl acetate and ethyl lactate.

## DISSCUSSION

Low-temperature fermentations produce wines with greater aromatic complexity. Nonetheless, the success of these fermentations greatly depends on the adaptation of yeast cells to cold. Changes in the plasma membrane composition have been directly related with the yeast adaptive response at different environmental temperatures in many studies 1,4,6,7. In our previous study 13, we screened most of the mutants of laboratory strain BY4742 encoding enzymes of the phospholipid, sterol and sphingolipid pathways in their growth capacity at 12°C. Those genes whose deletion showed growth impairment at low temperature were also deleted and overexpressed in the derivative haploid *ho*QA23. In this previous study, determination of growth parameters was carried out in minimal medium (SC) to avoid interferences of the other stresses exerted during wine fermentation (osmotic, pH, ethanol, etc.). Despite the many phenotypic differences observed between the laboratory and the commercial wine yeast strains, we detected some key lipid metabolism genes in promoting better growth at low temperature 13. We are, however, aware that these mutant and overexpressing strains with differential phenotypes at low temperature should be tested in an environment that mimics grape must fermentation. The aim of this study was to confirm the importance of these genes in growth and fermentation activity at low temperature by using a SM.

In this study, a SM without anaerobic factors was used to avoid the incorporation of some sterols and unsaturated fatty acids from the medium 1. If we compare the generation time of the mutants growing in SC 13 and SM (this study), phospholipid (∆*psd1* and ∆*opi3*) and sterol mutants (∆*erg3* and ∆*erg6*) showed strongly impaired growth at low temperature, regardless of the media. However, no difference in the growth of sphingolipid mutants in SM was noted. Likewise, similar results were observed when we analyzed the growth of the overexpressing strains growing in SC and SM at low temperature. The overexpressions of *OPI3*, *IDI1* and *LCB3 *produced a phenotype with better growth in both culture media at low temperature in comparison to the control strain. Unlike the results obtained in SC, the overexpression of *OLE1* in SM enhanced growth at low temperature. All these results demonstrate the importance of testing growth capacity in an environment that mimics grape must fermentation.

The analysis of fermentation performance showed that the mutants with worst growth at 12°C were unable to finish low-temperature fermentation (∆*psd1*, ∆*opi3, *∆*erg3* and ∆*erg6*). We also observed stuck fermentations at 28°C in the fermentation carried out by ∆*erg3* and ∆*erg6*. These genes are involved in the last steps of ergosterol biosynthesis, and their function must be crucial for growth in SM and fermentation activity because these strains were strongly affected in both activities, with minimum influence of fermentation temperature. The most specific response to low temperature was related to *PSD1*. Growth and fermentation performance were barely affected in the ∆*psd1 *and pGREG *PSD1* strains at optimum temperature. Nevertheless, these strains presented major phenotypic differences in comparison to the control performed in low temperature fermentations. *PSD1 *encodes a phosphatidylserine decarboxylase (Psd1p) of the mitochondrial membrane, which converts PS into PE. Recent works have related increases in PE or decreases in the PC/PE ratio as a general response to low temperature in different strains and species of *Saccharomyces* 7,15.

Another specific response at low temperature has also been observed in overexpressing strain pGREG *OLE1*, which showed improved fermentation performance and a shorter generation time than the control strain, as previously reported by 16.

The overexpression of two sphingolipid genes (*LCB3* and *DPL1*) improved fermentation activity at 12°C. The pGREG *LCB3 *strain also showed a shorter GT at low temperatures in comparison to the control strain. The *LCB3 *gene encodes a phosphatase that is capable of dephosphorylating long-chain bases, dihydrosphingosine-1-phosphate (DHS-1-P) and phytosphingosine-1-phosphate (PHS-1-P), and the *DPL1 *gene encodes a lyase, which cleaves the same long-base phosphates 17. Mandala *et al*. 18 demonstrated that ∆*lcb3 *and ∆*dpl1* dramatically enhanced survival upon severe heat shock. Conversely, our data evidence that the overexpression of these genes improves growth and fermentation performance at low temperature.

In our opinion, although the importance of these genes in yeast cold adaptation is quite conclusive, these data were obtained in a derivative haploid of an industrial strain and using SM. In an attempt to take another step forward to approach industrial conditions, we decided to overexpress the four genes showing a specific response at low temperature in the industrial strain QA23 to subsequently test these new overexpressing strains in both synthetic and natural grape musts. When using non integrative plasmids, gene overexpression requires the cultivation of overexpressing strains in the presence of antibiotics or in a chemically-defined medium in order to maintain the plasmid by selection pressure. We recently adapted a novel, efficient method of stable gene overexpression in the industrial wine strains of *S. cerevisiae* 14. This strategy is based on multi-copy chromosomal integration by homologous recombination with ubiquitous δ elements, which are integral parts of yeast transposons 19. These new overexpressing strains did not show major phenotypic differences in low-temperature fermentation if compared with industrial strain QA23. The different phenotype shown by the overexpressing strains constructed by the two different methods (chromosome integration and centromeric plasmid) could be explained by the different ploidy of parental strains or the different number of new copies of the target gene, which resulted in a lower overexpression levels in the overexpressing strains constructed by chromosome integration. Despite the minor differences observed, fermentation length was shorter for strains δ*OLE1*δ and δ*DPL1*δ if compared to commercial wine yeast QA23 both in "Parellada" and "Albariño" grape must fermentations.

As changes in the fatty acid profile can have a direct impact on aroma production 4, we analyzed the fermentative aroma compounds in the wines obtained with both the overexpressing strains and commercial wine yeast QA23. The overexpression of these genes did not lead to major modifications in the aroma profile of the final wines, except for the wines fermented by strain δ*OLE1*δ, which achieved a poorer production of several aroma compounds (ethyl lactate, isoamyl alcohol and ethyl acetate). Saerens *et al. *20 reported an indirect correlation between unsaturated fatty acids and ethyl acetate.

In summary, most of the results have supported the screening done in the previous study because the constructed mutants exhibited impaired growth and fermentation activity, whereas the overexpressing strains of these genes reduced the GT and fermentation length. Genes such as *DPL1, LCB3*, *OLE1* and *PSD1* have been seen to play a crucial role in cold adaptation, and the genetic manipulation of these genes may improve the performance of wine yeasts in low-temperature fermentations. Construction of overexpressing strains by chromosomal integration is a clean, safe method that can be used in the wine industry. Moreover we can increase the overexpression by integrating more copies of the target gene in successive rounds of transformations of the same commercial wine strain.

## MATERIALS AND METHODS

### Construction of mutant and overexpressing strains 

Most of the deleted mutant and the selected overexpressing strains were constructed in our previous work 13 in the background of a derivative haploid of commercial wine strain QA23 (*ho*QA23) (Lallemand S.A., Canada) 21. All the genes were deleted using the short flanking homology (SFH) method based on the *KanMX4* deletion cassette 22 and were overexpressed by cloning into the centromeric plasmid pGREG505, as described in 23. These genes are listed in Table 1. *IDI1*, *OLE1* and *CHO2* were overexpressed only because the deletion of the two former genes produced an unviable phenotype 24 and the deletion of *CHO2 *caused an auxotroph phenotype for choline in derivative wine strain *ho*QA23 13. The haploid QA23 strain transformed with empty plasmid pGREG505 (*ho*QA23-pGREG) was used as control of the overexpressing strains.

**Table 1:** List of the lipid genes used in this study.

**Standard name**	**Systematic name**	**Molecular function**	**Substrate**	**Product**
*PSD1*	YNL169C	Phosphatidylserine decarboxylase I	PS	PE
*CHO2*	YGR157W	Phosphatidylethanolamine N-Methyltransferase	PE	M-PE/ MM-PE
*OPI3*	YJR073C	Phospholipid methyltranferase	MM-PE	PC
*ERG3*	YLR056W	C-5 sterol desaturase	Episterol	5,7,24(28)-ergostatrienol
*ERG6*	YML008C	Sterol 24-C methyltransferase	Zymosterol	Fecosterol
*IDI1*	YPL117C	Isopentenyl-diphosphate delta-isomerase	Delta3-isopentenyl-PP	Dimethylallil-pyrophosphate
*LCB3*	YJL134W	Sphingosine-1-phosphate phosphatase	DHS-P, PHS-P	DHS, PHS
*LCB4*	YOR171C	D-erythro-sphingosine kinase	DHS, PHS	DHS-P, PHS-P
*DPL1*	YDR294C	Sphinganine-1-phosphate aldolase	DHS-P	Palmitaldehyde Phosphoryl-ethanolamine
*OLE1*	YGL055W	Stearoyl-CoA-desaturase	Saturated fatty acids	Unsaturated fatty acids

Moreover in this study, the stable overexpressing strains were constructed by integrating one or more copies of genes *DPL1*, *LCB3*, *OLE1* and *PSD1* into the genome of the commercial wine yeast strain QA23. To do this, the method proposed by 19 was followed with some modifications 14. This genetic transformation system allows the integration of the selected gene in the δ sequences of the *S. cerevisiae *genome. Briefly, *KanMX4* was integrated approximately 400 bp downstream of the stop codon of the gene of interest. After checking the correct integration of *KanMX4*, a new PCR product incorporating the gene of interest, with its own promoter and the gene of resistance to geneticin (*KanMX4*), was obtained. These PCR fragments were generated with primers D1-Forward and KanD2-Reverse, which contain homologous tails to the δ sequences of Ty 19. The expression cassettes for genes* DPL1*, *LCB3*, *OLE1* and *PSD1* were used to transform wine yeast strain QA23. Transformants were selected by geneticin resistance and PCR was used to test the correct insertion of the cassettes into the δ sequences. The new overexpressing strains were named δ*DPL1*δ, δ*LCB3*δ, δ*OLE1*δ and δ*PSD1*δ.

### Gene expression analysis by real-time quantitative PCR

Total RNA of 10^8 ^cell/ml was isolated as described by 25 and was resuspended in 50 µl of DEPC-treated water. Total RNA suspensions were purified using the High Pure Isolation kit (Roche Applied Science, Germany) according to the manufacturer’s instructions. RNA concentrations were determined using a NanoDrop ND-1000 spectrophotometer (NanoDrop Technologies, USA), and RNA quality was verified electrophoretically on a 0.8% agarose gel. Solutions and equipment were treated so that they were RNase-free, as outlined in 26.

Total RNA was reverse-transcribed with Superscript^TM^ II RNase H^-^ Reverse Transcriptase (Invitrogen, USA) in a GenAmp PCR System 2700 (Applied Biosystems, USA). The reaction contained 0.5 µg of Oligo (dT)_12-18_ Primer (Invitrogen, USA) and 0.8 µg of total RNA as a template in a total reaction volume of 20 µl. Following the manufacturer’s guidelines, after denaturation at 70°C for 10 min, cDNA was synthesized at 42°C for 50 min, and then the reaction was inactivated at 70°C for 15 min.

The primers were designed with the *Saccharomyces* Genome Database (SGD), except for housekeeping gene *ACT1*, which was previously described by 27. All the amplicons were shorter than 100 bp, which ensured maximal PCR efficiency and the most precise quantification. Real-Time Quantitative PCR was performed in LightCycler 480 SYBR Green I Master (Roche, Germany). The SYBR PCR reactions contained 2.5 µM of each PCR primer, 5 µl of cDNA and 10 µl of SYBR Green I Master (Roche, Germany) in a 20-µl reaction.

All the PCR reactions were mixed in a LightCycler 480 Multiwell Plate 96 (Roche, Germany) and cycled in a LightCycler 480 Instrument II, 96-well thermal cycler (Roche, Germany) under the following conditions: 95°C for 5 min, and 45 cycles at 95°C for 10 sec, at 55°C for 10 sec and 72°C for 10 sec. Each sample had two controls that were run in the same PCR: no amplification control (sample without reverse transcriptase reaction) to avoid interference by contaminant genomic DNA and no template control (sample with no RNA template) to avoid interference by primer-dimer formation. All the samples were analyzed in triplicate with the LightCycler 480 Software, version 1.5 (Roche, Germany) and the expression values were averaged. The gene expression levels are shown as a relative value in comparison to the control. Housekeeping gene *ACT1 *was used as an endogenous reference gene to normalize input amounts.

### Generation time

Growth was monitored at 600 nm in a SPECTROstar Omega instrument (BMG Labtech, Offenburg, Germany) at 12°C and 28°C, as described in 13.

Growth parameters were calculated from each treatment by directly fitting OD measurements versus time to the reparametized Gompertz equation proposed by 28:

**Figure:**
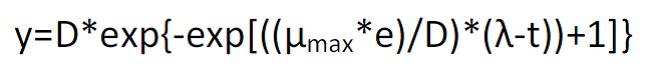


where y=ln(OD_t_/OD_0_), OD_0_ is the initial OD and OD_t_ is the OD at time t; D=ln(OD_t_/OD_0_) is the asymptotic maximum, µ_max_ is the maximum specific growth rate (h^-1^), and λ is the lag phase period (h) 29. The R code (statistical software R v.2.15 (R Development Core Team, 2013)) was used to fit the results to the reparametized Gompertz equation. Generation time was calculated using the equation t_d_=ln2/µ. Values were normalized by dividing, with its control, the generation time of strains* ho*QA23 or *ho*QA23-pGREG. Values lower than 1 indicated a shorter generation time, whereas values higher than 1 indicated a longer generation time as compared to the control.

### Fermentations

All strains were cultured in the SM (pH 3.3) described by 30, but with 200 g/L of reducing sugars (100 g/L glucose + 100 g/L fructose) and without anaerobic factors 27. The following were utilized: organic acids, malic acid 5 g/L, citric acid 0.5 g/L and tartaric acid 3 g/L; mineral salts KH_2_PO_4_ 750 mg/L, K_2_SO_4_ 500 mg/L, MgSO_4_ 250 mg/L, CaCl_2_ 155 mg/L, NaCl 200 mg/L, MnSO_4_ 4 mg/L, ZnSO_4_ 4 mg/L, CuSO_4_ 1 mg/L, KI 1 mg/L, CoCl_2_ 0.4 mg/L, H_3_BO_3_ 1 mg/L and (NH4)_6_Mo_7_O_24_ 1 mg/L; vitamins myo-inositol 20 mg/L, calcium pantothenate 1.5 mg/L, nicotinic acid 2 mg/L, chlorohydrate thiamine 0.25 mg/L, chlorohydrate pyridoxine 0.25 mg/L and biotin 0.003 mg/L. The assimilable nitrogen source used was 300 mg N/L (120 mg N/L as ammonium and 180 mg N/L in the amino acid form). Geneticin was also added (200 mg /L) to the SM of the overexpressing strains to ensure plasmid stability.

The overexpressing strains constructed by chromosomal integration were also cultured in two natural grape musts: "Albariño" grape must, which contained about 200 g/L of reducing sugars (100 g/L glucose + 100 g/L fructose); "Parellada" grape must, which contained about 180 g/L of reducing sugars (90 g/L glucose + 90 g/L fructose). Prior to inoculation, the grape must was treated with 1 ml/L of Velcorin (trade name for dimethyldicarbonate; Merck, Hohenbrunn, Germany). The use of this antimicrobial agent resulted in the practical elimination of the microbiota of the NM, tested by plating the grape must on YPD plates and incubated for 72 h at 30°C.

In the fermentations performed by the mutant and overexpressing strains of *ho*QA23, and also in those performed in "Albariño" by the overexpressing strains of QA23, the inoculated population came from an overnight culture in YPD at 30°C. In order to avoid other stresses (osmotic, pH, etc.) to the inoculum produced by changing from YPD to grape must, the fermentations carried out by the stable overexpressing strains of QA23 in SM and NM "Parellada" were inoculated with the cells from an overnight culture at 30°C in the same fermentation media. After counting microscopically, the appropriate dilution of the overnight culture was transferred to the grape must to achieve an initial cell concentration of 2 x 10^6^ cells/ml.

Fermentation activity of the mutant and overexpressing strains of *ho*QA23 were tested at 28°C and 12°C, and fermentation activity of stable overexpressing strains were analyzed only at 12°C. Fermentations were performed with continuous orbital shaking at 100 rpm. Fermentations were carried out in laboratory-scale fermenters using 100-ml bottles filled with 60 ml of media, which were fitted with closures that enabled carbon dioxide to escape and samples to be removed. Yeast cell growth was determined by absorbance at 600 nm and by plating samples at the end of fermentation on YPD agar at an adequate dilution to be incubated for 2 days at 30°C. Fermentation was monitored by measuring the density of the media (g/L) using a Densito 30 PX densitometer (Mettler Toledo, Switzerland). Fermentation was considered to have been completed when density was below 998 g/L. Residual sugars were also determined by HPLC in a Surveyor Plus Chromatograph (Thermo Fisher Scientific, Waltham, MA, USA).

### Volatile aroma compounds

Higher alcohols and esters were analyzed based on a headspace solid-phase microextraction (SPME) technique using a 100 µm poly-dimetylsiloxane (PDMS) fiber (Supelco, Sigma-Aldrich, Spain). Aliquots of 1.5 ml of the sample were placed into 15 ml vials and 0.35 g of NaCl and 20 µl of 2-heptanone (0.005%) was added as an internal standard. Vials were closed with screwed caps and 13 mm silicone septa. Solutions were attired for 2 h to obtain the required headspace-liquid equilibrium. Fibers were injected through the vial septum and exposed to the headspace for 7 min to then be desorbed for 4 min in a gas chromatograph (TRACE GC Ultra, Thermo Scientific), with a flame ionization detector (FID) equipped with an HP INNOWax 30 m x 0.25 mm capillary column coated with a 0.25 m layer of cross-linked polyethylene glycol (Agilent Technologies). The carrier gas was helium (1 ml/min) and the oven temperature program utilized was: 5 min at 35°C, 2°C/min to 150°C, 20°C/min to 250 °C. The injector and detector temperatures were maintained at 220°C and 300°C respectively. A chromatographic signal was recorded by the ChromQuest program. Volatiles compounds were identified by comparing the retention time for reference compounds. Volatile compound concentrations were determined using calibration graphs of the corresponding standard volatile compounds.

### Statistical data processing

All the experiments were repeated at least 3 times. Data are reported as the mean value ± SD. Significant differences among the control strain, the mutant and the overexpressing strains were determined by *t*-tests (SPSS 13 software package, USA). The statistical level of significance was set at P ≤ 0.05. A principal component analysis was done using the vegan package (rda function) of the statistical software R, v.2.15 31.
